# A predictive model based on random forest for shoulder-hand syndrome

**DOI:** 10.3389/fnins.2023.1124329

**Published:** 2023-03-31

**Authors:** Suli Yu, Jing Yuan, Hua Lin, Bing Xu, Chi Liu, Yundong Shen

**Affiliations:** ^1^Department of Hand and Upper Extremity Surgery, Jing’an District Central Hospital, Fudan University, Shanghai, China; ^2^Department of Geriatric Rehabilitation Medicine, Shanghai Fourth Rehabilitation Hospital, Shanghai, China; ^3^Department of Geriatrics Center, National Clinical Research Center for Aging and Medicine, Jing’an District Central Hospital of Shanghai, Fudan University, Shanghai, China; ^4^Department of Hand Surgery, Huashan Hospital, Fudan University, Shanghai, China

**Keywords:** ensemble learning, random forest, shoulder-hand syndrome, stroke rehabilitation, predictive model

## Abstract

**Objectives:**

The shoulder-hand syndrome (SHS) severely impedes the function recovery process of patients after stroke. It is incapable to identify the factors at high risk for its occurrence, and there is no effective treatment. This study intends to apply the random forest (RF) algorithm in ensemble learning to establish a predictive model for the occurrence of SHS after stroke, aiming to identify high-risk SHS in the first-stroke onset population and discuss possible therapeutic methods.

**Methods:**

We retrospectively studied all the first-onset stroke patients with one-side hemiplegia, then 36 patients that met the criteria were included. The patients’ data concerning a wide spectrum of demographic, clinical, and laboratory data were analyzed. RF algorithms were built to predict the SHS occurrence, and the model’s reliability was measured with a confusion matrix and the area under the receiver operating curves (ROC).

**Results:**

A binary classification model was trained based on 25 handpicked features. The area under the ROC curve of the prediction model was 0.8 and the out-of-bag accuracy rate was 72.73%. The confusion matrix indicated a sensitivity of 0.8 and a specificity of 0.5, respectively. And the feature importance scored the weights (top 3 from large to small) in the classification were D-dimer, C-reactive protein, and hemoglobin.

**Conclusion:**

A reliable predictive model can be established based on post-stroke patients’ demographic, clinical, and laboratory data. Combining the results of RF and traditional statistical methods, our model found that D-dimer, CRP, and hemoglobin affected the occurrence of the SHS after stroke in a relatively small sample of data with tightly controlled inclusion criteria.

## Introduction

Complex regional pain syndrome (CRPS) (Harden et al., 2007) are neuropathic pain disorders that generally affect the extremities and can occur after myocardial infarction, cervical spondylosis, craniocerebral trauma, and cerebrovascular disease (Shaparin et al., 2014). CRPS of the paralyzed upper limb after stroke is frequently called shoulder-hand syndrome (SHS), also known as reflex sympathetic dystrophy (RSD), and classified as CRPS type I by the International Association for the Study of Pain.

The etiology of SHS is still unclear (Bussa et al., 2015), and the prevalence varies greatly. Researchers in Korea reported the incidence rate ranged from 2 to 50% (Kim et al., 2020), and the Chinese reported from 12.5 to 74.1% (Zhong and Tang, 2009). SHS usually occurs 1−6 months after a cerebrovascular accident, which happens to be the period with the highest potential for rehabilitation (Kumar et al., 2009) and usually has a significant impact on the patient’s life quality and functional recovery. The paralyzed upper limbs frequently appeared painful, and edematous. The symptoms usually started from the hands, then often spread to the fingers and palms, and in severe cases to the lower forearms. Sensory disturbances including burning, stiffness, sweating, cold, or fever occurred along with the nerve distribution and the areas of injury. The pain usually increased with hand joint movements (especially passive movement). If not treated and controlled in time, long-term immobilization and relative hypoxia of tissues would cause atrophy of interosseous muscles and lumbricals, contracture of hand joints, especially metacarpophalangeal capsules, together with fibrosis of exudates causing adhesions, thickening of synovial bursae, and changes of corresponding joint bones, result in irreversible disability (Forouzanfar et al., 2002; Pertoldi and Di Benedetto, 2005; Hartwig et al., 2012; Pervane Vural et al., 2016).

The diagnosis of CRPS is often difficult due to the lack of confirmatory tests, and SHS’s diagnosis is not specific and more complex than in other pathological situations. SHS is now generally believed to be associated with incorrect movement patterns in the early stages of stroke patients resulting in shoulder and wrist injuries, impaired upper extremity fluid return, and vasomotor dysfunction following central nervous system injury (Lee et al., 2021). Stroke patients usually have arms hanging to their side for long periods in the recumbent and seated positions. The wrist joint is flexed, the shoulder girdle is retracted and sunken, and the forearm is internally rotated. The flexion and compression of the wrist joint can block a venous return to the upper extremity, resulting in swelling of the wrist and forearm (especially in the fingers and wrist) (Geurts et al., 2000). It is internationally recognized that the increased sympathetic excitability and decreased muscle strength of the affected limb after central nervous system injury cause the muscles to lose their “muscle pump” effect, while the obstruction of a venous return due to motor dysfunction leads to edema and pain in the hemiplegic upper limb (Zorowitz et al., 1996). Currently, no guidelines for the prevention of SHS have been established. Treatments include non-pharmacological therapy, pharmacological therapy, regional anesthesia, neuromodulation, sympathectomy, auxiliary compression facilities (Lin et al., 2020), also rehabilitation exercises. Currently, there is no single treatment to be universally effective. Since it was first introduced 70 years ago, it still is challenging work to make early detection of SHS. Our work aims to establish a predictive model of SHS by machine learning algorithm based on patient clinical information to highlight the risk factors, make early diagnoses, and detect potential intervention targets.

Random forest (RF) is an ensemble learning algorithm to predict a binary outcome (classifier) or a numerical value (regressor). It utilized bootstrap aggregating of both sample and feature bagging to create an uncorrelated forest of decision trees. The method is useful when the samples are relatively small (Breiman, 1996, 2001; Deo, 2015). In RF classification, many classification and regression trees (CARTs) are generated with bootstrap’s resampling technique repeatedly and randomly select m samples from the original training sample set of N (m < N).

In the process of generating a tree, feature selection is needed for splitting. The splitting principle is to improve the purity as much as possible, which can be measured by indicators such as information gain, gain rate, and Gini index. The bootstrap method is also applied for randomly selected parts of the features to find the one that makes the smallest Gini index, and the optimal solution is found among the selected features and applied to the nodes for splitting (Breiman, 2001; Kuhn and Johnson, 2013; Bzdok et al., 2018; Vabalas et al., 2019).

Random forest makes it easy to evaluate variable importance, or contribution, to the model. There are a few ways to evaluate feature importance. Gini importance and mean decrease in impurity (MDI) are usually used to measure how much the model’s accuracy decreases when a given variable is excluded. However, permutation importance, also known as mean decrease accuracy (MDA), is another important measure. MDA identifies the average decrease in accuracy by randomly permutating the feature values in out-of-bag samples (Breiman, 2001; Kuhn and Johnson, 2013; Bzdok et al., 2018; Vabalas et al., 2019). These methods allow us to measure the role of each feature in the classification and thus the importance of the occurrence of the disease.

This study intends to initially explore the application of RF to establish a predictive model and measure the importance of each variation in classification.

## Materials and methods

### Patient selection

This study included all adult patients (aged 18 years and older) who were hospitalized at the rehabilitation department during December 2020 and June 2021 in Shanghai Fourth Rehabilitation Hospital.

**FIGURE 1:**
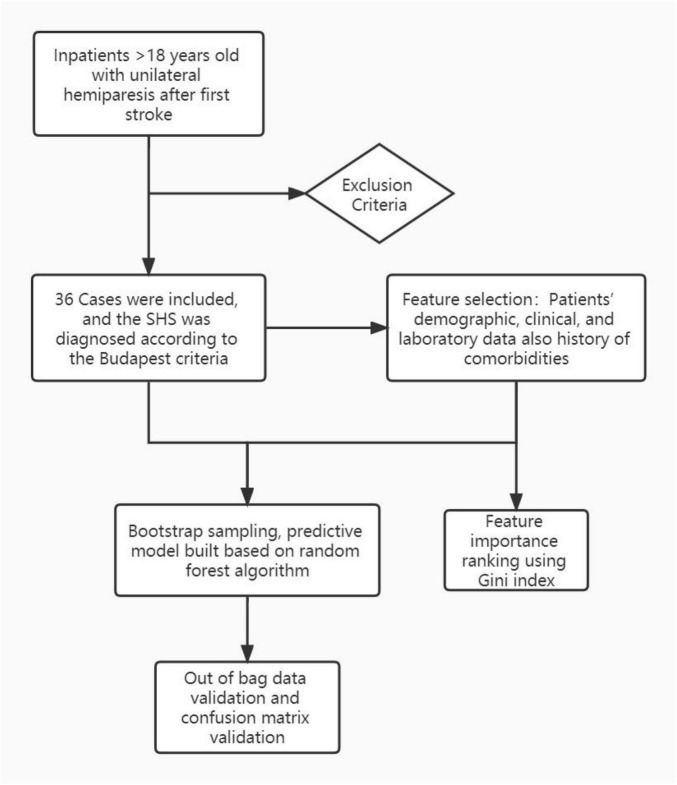
The flow chart of the experiment protocol.

We included first-onset stroke patients with unilateral hemiparesis, who were admitted to our rehabilitation department within 1 month after stabilization of neurological symptoms. Exclusion criteria: subjects with more than one stroke episode, patients with a history of surgery on the affected upper limb, combined traumatic brain injury, spinal cord injury, acute myocardial infarction, heart failure, history of tumor, and subjects with severe liver and kidney dysfunction, Subjects with combined immune system disorders, hematologic disorders, subject with fever and pulmonary infections at the time of admission (see Figure 1).

Since this study was a predictive model establishment, the laboratory data were collected before the occurrence of the SHS. The site of the stroke was clarified by brain computed tomography or magnetic resonance imaging in all cases. All patients received standardized functional rehabilitation training after admission.

### Ethics approval

This study was approved by the Institutional Review Board of Shanghai Fourth Rehabilitation Hospital with a waiver of informed consent due to the retrospective nature of the study. The data of this study are available from the corresponding author upon reasonable request.

### Assessed variables and feature selection

Patients’ demographic, clinical, and laboratory data were used as possible variables as well as the history of the patient’s comorbidities. Demographic data included patients’ age and sex and laboratory findings consisted of routine examination of blood and biochemistry (Hemoglobin, white blood cell count, platelet count, C-reactive protein, 25-OH vitamin D, D-dimer, creatinine, B-type brain natriuretic peptide, urine microalbumin, homocysteine). Clinical variables included stroke type (infarction, hemorrhage, infarction combined with hemorrhage) and lesion location (cerebral cortex, cerebellum, thalamus, basal ganglia, brainstem).

Post-operative function assessments were not assessed in this study, Due to differences in scale selection and quality control across patients. The outcome was assessed with a follow-up of 6 months of rehabilitation employing the Budapest criteria (Table 1)–the mainstream diagnostic tool for CRPS (Pergolizzi et al., 2018).

**TABLE 1:** The Budapest criteria: In order to make a clinical diagnosis of CRPS, the following four criteria must be met (Pergolizzi et al., 2018).

No	Criteria	Categories			
		Sensory	Vasomotor	Sudomotor/edema	Motor/trophic
1	Continuing pain, disproportionate to any inciting event	–	–	–	–
2	Symptoms: must report at least one symptom in three of the four categories shown to the right	Hyperesthesia; Allodynia	Temperature asymmetry; changes in skin color; skin color asymmetry	Edema; sweating changes; sweating asymmetry	Decreased range of motion; motor dysfunction; trophic changes (hair, nails, skin)
3	Signs: at the time of evaluation, must have at least one sign in two or more of the categories shown to the right	Hyperalgesia (pinprick); Allodynia (light touch or temperature); deep somatic pressure; joint movement	Skin temperature asymmetry (>1°C); changes in skin color; skin color asymmetry	Edema; sweating changes; sweating asymmetry	Decreased range of motion; motor dysfunction (weakness, tremor, dystonia); trophic changes (hair, nails, sin)
4	No other diagnosis can better explain the patient’s signs and symptoms	–	–	–	–

### Data analysis

Data analysis was performed in python version 3.2.8., using the NumPy, Pandas, scikit-learn, scipy, and matplotlib modules.

Data were summarized by the sample mean and standard deviation (SD) for a continuous variable and by the count for a categorical variable. Demographic characteristics, comorbidities, and laboratory data were compared between two groups using two independent-sample *t*-tests for continuous variables and Fisher’s exact tests for categorical variables. Statistically, the significant difference was denoted by a *P*-value of less than 0.05, whereas practical significance was represented by effect sizes. Cohen’s d was used to measure effect sizes. In general, a d of 0.2 or smaller is considered to be a small effect size, a d of around 0.5 is considered to be a medium effect size, and a d of 0.8 or larger is considered to be a large effect size.

### Data pre-processing

Empty data were replaced, and the missing value was filled in with the mean to complete the information of all cases. Variables were classified into numeric and categorical variables according to their type.

The categorical variables were:

Gender (male 1, female 2); whether lacunar infarction (yes 1, no 0); brain (lesion involved 1, not involved 0), cerebellum (lesion involved 1, not involved 0), thalamus (lesion involved 1, not involved 0), basal ganglia (lesion involved 1, not involved 0), brain stem (lesion involved 1, not involved 0), stroke type (infarction 1, hemorrhage 2); hemiplegic limbs (left side 1, right side 2); hypertension (d 1, not combined 0), atrial fibrillation (combined 1, not combined 0), diabetes (combined 1, not combined 0).

Numerical variables were: age, white blood cell count, hemoglobin, platelet count, C-reactive protein (CRP), B-type brain natriuretic peptide, D-dimer, 25-OH vitamin D (25-OHD), D dimer Body, creatinine, homocysteine, urine microalbumin.

Use one-hot encoding to convert categorical variables into numeric variables. Perform normalization (scaling) processing for numerical variables. The label prediction was set to 0 (SHS does not occur) and 1 (SHS occurs).

### Random forest algorithm establishment

To predict the occurrence of SHS with imbalanced data recorded (more patients with SHS than without SHS), a classified RF algorithm was trained. Training data were gathered by repeated subsampling (bootstrapping) for inclusion in each tree. Data excluded from the bootstrap subsample (approximately 30% for each tree) were called out-of-bag (OOB). These data were further aggregated into the OOB sample. The number of included decision trees was optimized to achieve the lowest possible error rate, which prevented the over-fitting of the trained model.

Variables were selected using the nested cross-validation and Gini index criterion (a measurement of node purity, the smaller the Gini index, the purer the node, and the more likely the split will take place). The number of trees was selected to minimize the OOB error rate. Node size was optimized using the same criterion.

Predictive power was evaluated on the corresponding OOB data. A confusion matrix was performed to calculate the sensitivity, specificity, and precision. OOB accuracy rate was calculated and receiver operating characteristic (ROC) curves were constructed with corresponding values of area under the curve (AUC) calculated. Values of AUC close to 1 suggest strong predictive capability, whereas values near 0.5 means poor prognostic power.

The RF algorithm had a great quality to measure the relative importance of each feature in the prediction. scikit-learn measured a feature’s importance by looking at how much the tree nodes that used that feature reduce impurity across all trees in the forest. The sum of all feature importance scores was equal to one.

Using the bootstrap method sampled 70% size as the training set and the remaining 30% as the test set. Use Python version 3.2.8 to call the Randomforestclassifier module in the scikit-learn library. A total of 99 decision trees were trained under default parameter values (n_estimators = 10, max_depth = None, min_samples_split = 2, random_state = 0). The score function was called to calculate the accuracy of the model on the test set. Confusion_matrix was to calculate the sensitivity, specificity, and precision of the model. The roc_curve function was to draw the ROC curve and obtain the AUC value to evaluate the predictive ability of the model. Feature_importance_ was to obtain the feature importance value and sort it according to its importance.

## Results

### Characteristics of the patients

The inclusion criteria were met by 36 patients (19 men and 13 women). The average age of patients was 59.23 ± 18.27 years. The main recorded comorbidities included arterial hypertension in 31 patients (86.11%), diabetes mellitus (regardless of the type) in 21 patients (58.33%), and atrial fibrillation in 8 patients (22.22%). Left hemiparesis was present in 16 patients (44.44%). Characteristics of the Patients were summarized in Table 2.

**TABLE 2:** Comparison data of demographic and clinical characteristics between the occurrence group and the non-occurring group.

	Occurrence	Non-occurring	*T*-value	Cohen’s d
Age	75.26 ± 10.43	73.35 ± 8.38	0.551815161	0.1
White blood cell	6.40 ± 1.94	7.22 ± 3.26	0.373135738	0.15
Hemoglobin	117.89 ± 20.57	130.82 ± 16.86	**0.048424703**	0.69
Platelet	209.53 ± 58.81	223.29 ± 82.35	0.564450443	0.19
C-reactive protein	11.41 ± 21.57	13.25 ± 14.86	0.77005131	0.1
B-type brain natriuretic peptide	78.77 ± 80.48	107.96 ± 169.24	0.523183027	0.22
D-dimer	1.80 ± 1.59	0.92 ± 0.77	**0.042652041**	0.7
25-OH vitamin D	30.86 ± 11.95	33.06 ± 12.79	0.596054774	0.18
Homocysteine	41.68 ± 25.78	86.65 ± 211.26	0.39585193	0.3
Creatinine	64.72 ± 13.79	67.20 ± 28.1	0.744649236	0.11
Urine microalbumin	54.16 ± 50.2	44.29 ± 43.16	0.533938047	0.21
			**Total case ratio**	**χ**
Sex male: female	12: 07	11: 06	23 (1): 13 (2)	0.99998918
Stroke type infarction: hemorrhage	16: 03	16: 01	32 (1): 4 (2)	0.925757
Hemiplegic later left: right	11: 08	5: 12	16 (1): 20 (2)	0.56656694
Without hypertension consolidation: non-consolidation	17: 02	14: 03	31 (1): 5 (0)	0.98405107
Without diabetes consolidation: non-consolidation	7: 12	14: 03	21 (1): 15 (0)	0.10544849
Without atrial fibrillation consolidation: non-consolidation	5: 14	3: 14	8 (1): 28 (0)	0.98327996

### Prediction of occurrence of post-stroke SHS

**FIGURE 2:**
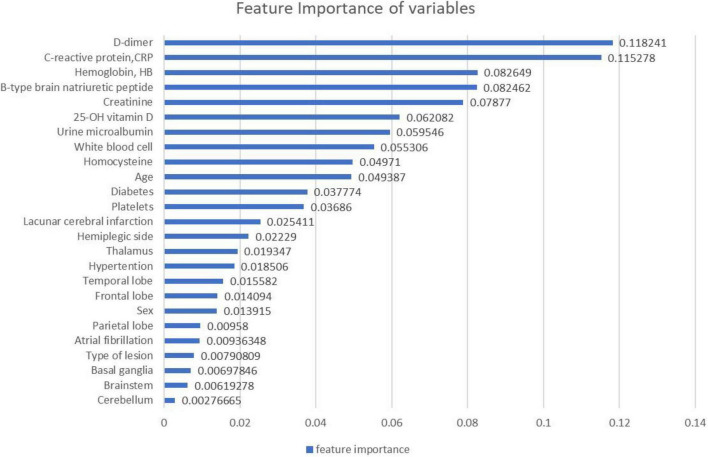
Feature importance ranking.

**FIGURE 3:**
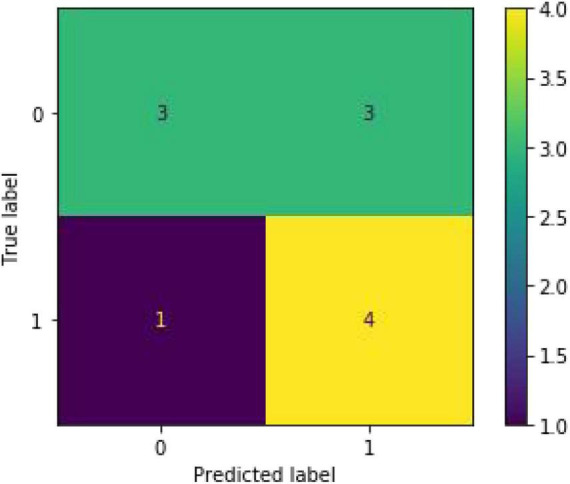
Confusion matrix analysis of the test set. (Accuracy: 0.875, sensitivity: 0.8, specificity: 0.5 and precision: 0.57142857).

**FIGURE 4:**
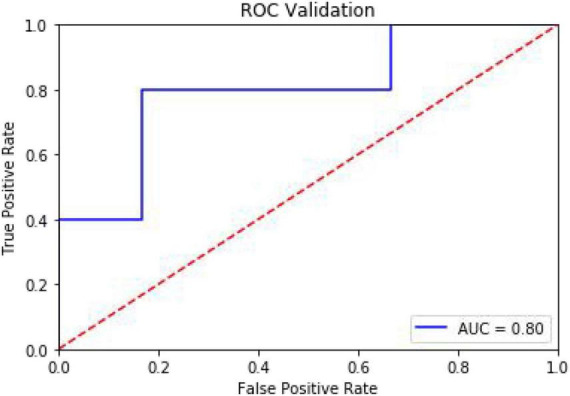
The area under the ROC curve evaluates the predictive ability of the model.

To predict whether the occurrence of SHS or not, classified RFs were constructed using 99 trees. The RF prediction model achieved an accuracy rate of 72.73% (0.7272727272727273) on OOB data. The two independent-sample *t*-tests were used for normally distributed and continuous data (see Table 2). The homoscedastic checks (*F*-value) were conducted for all data. The results showed there was a statistically significant difference between the two groups in terms of hemoglobin (*p* = 0.048424703) and D-dimer (*p* = 0.042652041). Then Cohen’s d was calculated to measure the effect sizes of the two terms. Cohen’s d for hemoglobin was 0.69, and for D-dimer was 0.70, both indicating medium differences between the groups. The occurrence group was prone to lower hemoglobin content and higher D-dimer values. The ranking of the feature importance ordered from largest to smallest was listed in Figure 2. Combining feature importance ranking with *t*-test results, our study indicated that D dimer, CRP, and HB played more important roles in classification and discrimination. The OOB accuracy rate of this model was 72.73%. The confusion matrix analysis (Figure 3) showed a model sensitivity of 0.8, specificity of 0.5, and precision of 0.57142857. The OOB ROC curve of the constructed model had an AUC of 0.8 (Figure 4).

Based on the above 25 characteristics, feature importance showed that the location of the lesion and hemiplegic side had little effect on the classification, indicating its less significant role in predicting the occurrence of SHS. Among the concomitant diseases, concomitant diabetes was more significant than hypertension and atrial fibrillation in predicting the development of SHS despite the low importance ranking score.

## Discussion

Shoulder-hand syndrome is the third most common complication of stroke, after falls and confusion. A previous study found the healthcare utilization cost after diagnosis of SHS is 2.17-fold increased, and at least 8 years after diagnosis such increase persisted (Elsamadicy et al., 2018).

The pathogenesis of SHS is not clear, risk factors cannot be identified, and there are no effective interventions available. Instead of the traditional statistics method, we applied a machine-learning algorithm to build a predictive model and identify high-risk factors for the occurrence of SHS.

Medical dataset may contain noisy information, missing and unbalanced data. When it comes to building disease prediction and diagnostic models, the relevant data may involve many features and complex and non-linear relationships between variable which are often beyond the capacity of traditional statistical data processing.

Random forest is a flexible and easy-to-use machine learning algorithm that hold the advantage of identify the pattern in medical dataset that may not be directly apparent, even without prior knowledge (Hostettler et al., 2018) which made it suitable for the medical dataset.

It can process very high dimensional data (many features) and without dimensionality reduction and feature selection. Also it can determine the importance of features and the interaction between different features. These advantages make it particularly suitable for the prediction of diseases with multifactorial involvement (such as SHS) in their pathogenesis, it enable all related or unrelated information during analysis. Also in terms of model building, it is not easy to over-fitting; It can balance the error for unbalanced data sets; If a large part of the features is missing, the accuracy can still be maintained (Vabalas et al., 2019; Liu et al., 2020; Yang et al., 2020).

The onset and severity of SHS appear to be related to the etiology of the stroke (Geurts et al., 2000; Su et al., 2021). Injurious stimuli from stroke lesions can induce inflammatory responses (Shaparin et al., 2014), including classical inflammation (an exaggerated inflammatory response and some chemical mediators around the primary afferent fibers induced peripheral sensitization), neurogenic inflammation (a localized neurogenic inflammation brought edema, vasodilation, and hyperhidrosis, or repeated discharge of the C fibers caused an increased central sensitization), impairment of the autonomic nervous system, and changes of the central nervous system (especially the reorganization of primary somatosensory cortex). Therefore, we included inflammation-related indicators, such as CRP and WBC, and other stroke-related biochemical indicators. Patients’ age, hemiplegia side, location of the lesion, etiology of stroke, and comorbidities were also selected into our feature. Feature Selecting for building predictive models is readily available for any medical institution which makes the model practical and generalizable.

It has been suggested that the key to the treatment of SHS is prevention, so some studies have focused on the risk factors for SHS. Potential risk factors had been recognized for SHS like being female, left hemiparesis, spasticity, shoulder subluxation, a lower Brunnstrom stage of the distal upper limb, and an inferior Barthel index (Braus et al., 1994; Sandroni et al., 2003; Ringman et al., 2004; Altas et al., 2020; Su et al., 2021). But no consensus was reached. A population-based study conducted by Sandroni et al. (2003), confirmed female patients had a higher incidence of CRPS compared with male patients, although the mechanisms were not clear so far. And some researchers indicated that patients with left paralysis were more subject to CRPS due to hemineglect more often occurring in a right hemispheric stroke (Braus et al., 1994; Ringman et al., 2004; Altas et al., 2020). Su et al. (2021) conducted a meta-analysis comprising 2,225 participants and claimed that age, side of the lesion, etiology of the stroke, the Brunnstrom arm stage, the duration of stroke, and shoulder pain were not found to be associated with SHS. But in *post hoc* analysis they found that women and paralysis of left limbs were found to be more likely to be the feature of SHS.

Our predictive model used feature importance to weigh each feature in classification. It assigned the score of input features based on their significance to predict the output. The more the features were responsible to predict the output, the more their score.

The feature importance of our model assigned a relatively greater value for D-dimer and hemoglobin. Also, statistics analysis found that the SHS group had higher D-dimer levels and lower hemoglobin levels. So a reasonable hypothesis arose whether anticoagulation drugs to decrease the D-dimer and therapies to increase the hemoglobin could be effective precautions or strategies for SHS. Oral corticosteroids were the only recommended drugs for SHS with level 1 evidence of the purpose of anti-inflammatory (Harden et al., 2007). This suggested the important role of anti-inflammatories in the prevention and treatment of SHS. The feature importance ranking assigned CRP a relatively high score in the classification which was not detected in the conventional statistical methods. This result corroborated the reliability and validity of the model to some extent. It also demonstrates the ability of machine learning to far outperform traditional statistical methods in the identification of high-risk factors, even on small sample data.

It is important to note that steroid therapy is somewhat limited in stroke patients (Long and Dagogo-Jack, 2011; Oray et al., 2016). So new approaches are warranted to solve this situation. For the possible implications of D-dimer, hemoglobin in the pathogenesis and treatment of SHS, such as anticoagulant therapy and therapeutic measures to increase hemoglobin levels may prevent or even treat SHS. Confirmation requires larger clinical trials and multiple-center cooperation to validate our preliminary results on a broader sample of numbers and sources.

We also recognize that our model had some limitations due to the small sample for modeling:

1. Our data came from a single medical institution, so our model and findings may not generalize to other populations.

2. The sample size in this study was small, that was why the ROC curve demonstrated a stepped shape. Although it was able to obtain better results with the bootstrap sampling method, it was not sufficient to train a fully valid prediction model and may not provide reliable discrimination of results for out-of-training data. The sample size should be further expanded in future work.

3. Feature selection lacked function assessing indicators. Some researchers indicated that lower function assessment of upper limb like (Barthel index, Brunnstrom stage) may be associated with SHS. The difficulty of our research to incorporate function scale was the lack of uniform quality control. There were inconsistent treatment strategies for functional assessment because patients were seen in different hospitals at the time of stroke onset. Some hospitals had early interventions for functional rehabilitation, while others did not. And there were inconsistencies in the time of assessment, and the functional scores of patients fluctuated widely without good certainty. So we discarded the corresponding functional indicators. Since this study was a preliminary study, we will establish uniform standards for the quality control of functional assessment in the follow-up study.

## Conclusion

The present study demonstrated a ensemble learning method using RF algorithm to predict the occurrence of SHS. Our findings highlighted the predictability of the onset of SHS using common and easily accessible metrics such as the blood biochemistry indicators, site of stroke, etiology, and concurrent diseases. The prediction model had an area under the ROC curve of 0.8, indicating considerable predictive ability. This method has the potential for early diagnosis and identification of high-risk factors with good utility.

## Data availability statement

The original contributions presented in this study are included in the article/Supplementary material, further inquiries can be directed to the corresponding authors.

## Ethics statement

The studies involving human participants were reviewed and approved by the Institutional Review Board of Shanghai Fourth Rehabilitation Hospital. The ethics committee waived the requirement of written informed consent for participation.

## Author contributions

SY, JY, and HL: data curation, formal analysis, study design, data collection, and writing–original draft. BX and CL: data collection and data curation. SY, HL, and YS: funding acquisition, supervision, writing–review and editing, and project administration. All authors contributed to the article and approved the submitted version.
