# Supplementary figures and images for: A predictive model based on random forest for shoulder-hand syndrome
Source: Front Neurosci. 2023 Mar 31;17:1124329. doi: 10.3389/fnins.2023.1124329 (PMC10102379; doi:10.3389/fnins.2023.1124329)

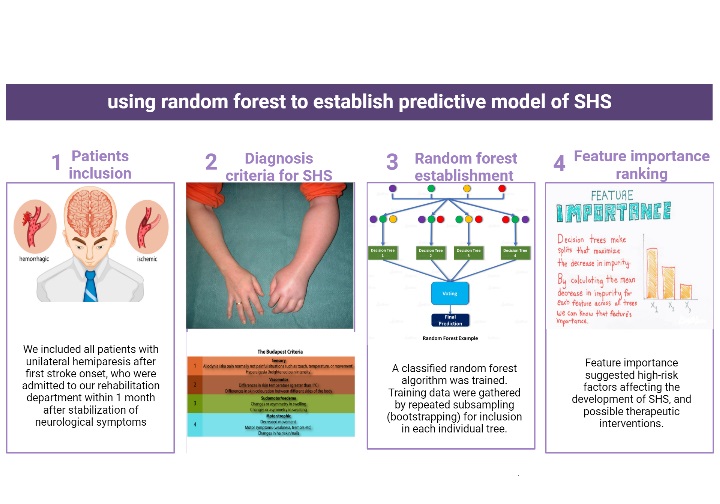

Supplement: Supplementary file 1 [file Image_1.JPEG]
